# Dr. Deckmann

**Published:** 1838-07

**Authors:** 


### DR. DECKMANN, OF KIEL.

C. G. Deckmann was born at Rendsburg, April 8, 1798, of parents in the lower class of society. He early distinguished himself at school and at the university. He commenced his medical studies at Kiel, and prosecuted them still further at Copenhagen. He returned from Copenhagen to Kiel, and, in March, 1824, took his degree of doctor, publishing a thesis under the following title: "Notæ quædam chemici præcipuè argumenti in aquas ophthalmicas." He then established himself in practice in the city of Schleswig, where he enjoyed good practice and the consideration of all his brethren, until 1829, when he was appointed extraordinary professor of anatomy and surgery in the university of Kiel. He published, in 1830, an interesting prospectus to his course of lectures, under the following title: "Studium Anatomiæ et Physiologiæ omnibus singularum artium cultoribus probat et ad prælectiones anthropologicas invitat C. G. Deckmann." Dr. Fischer having died in 1832, Deckmann was nominated ordinary professor, and took charge of the surgical clinic in the Frederick's Hospital. Of this clinic he published interesting reports in "Pfaff's Mittheilungen" for 1833. While in the flower of his age, and in high and increasing reputation, he was attacked with hæmoptysis and phthisis, which carried him off, after long suffering, on the 25th February, 1837, in the thirty-ninth year of his age. Professor Deckmann was an excellent surgeon and teacher, and most faithful and zealous in the performance of all his duties.†

† From a notice, by Dr. Pfaff, in Sachs's Almanach, for 1838.

